# Definition and management of varicella zoster virus-associated meningoradiculitis: a case report

**DOI:** 10.1186/s13104-016-2257-2

**Published:** 2016-09-26

**Authors:** Vincent Luisier, Lalensia Weber, Daniel Fishman, Gérard Praz, Joseph-André Ghika, Didier Genoud, Joelle Nsimire Chabwine

**Affiliations:** 1 Internal Medicine Division, Valais Hospital, Avenue Grand Champsec, 80, 1951 Sion, Switzerland; 2 Emergency Department, Valais Hospital, Avenue Grand Champsec, 80, 1951 Sion, Switzerland; 3 Infectious Disease Division (Central Institute), Valais Hospital, Avenue Grand Champsec, 80, 1951 Sion, Switzerland; 4 Division of Neurology, Valais Hospital, Avenue Grand Champsec, 80, 1951 Sion, Switzerland; 5 Neurology Unit, Department of Medicine, Faculty of Sciences, University of Fribourg, Chemin du Musée 5, 1700 Fribourg, Switzerland

**Keywords:** Varicella zoster virus, Meningoradiculitis, Herpes, Zoster, Paresis, Cell-mediated immunity

## Abstract

**Background:**

The varicella zoster virus affects the central or peripheral nervous systems upon reactivation, especially when cell-mediated immunity is impaired. Among varicella zoster virus-related neurological syndromes, meningoradiculitis is an ill-defined condition for which clear management guidelines are still lacking. Zoster paresis is usually considered to be a varicella zoster virus-peripheral nervous system complication and treated with oral antiviral therapy. Yet in the literature, the few reported cases of herpes zoster with mild cerebral spinal fluid inflammation were all considered meningoradiculitis and treated using intravenous antiviral drugs, despite absence of systemic signs of meningitis. Nevertheless, these two clinical pictures are very similar.

**Case presentation:**

We report the case of an alcohol-dependent elderly Caucasian man presenting with left lower limb zoster paresis and mild cerebral spinal fluid inflammation, with favorable outcome upon IV antiviral treatment. We discuss interpretation of liquor inflammation in the absence of clinical meningitis and implications for the antiviral treatment route.

**Conclusion:**

From this case report we suggest that varicella zoster virus-associated meningoradiculitis should necessarily include meningitis symptoms with the peripheral neurological deficits and cerebral spinal fluid inflammation, requiring intravenous antiviral treatment. In the absence of (cell-mediated) immunosuppression, isolated zoster paresis does not necessitate spinal tap or intravenous antiviral therapy.

## Background

Varicella zoster virus (VZV) is an exclusively human virus primarily causing chickenpox [1]. After a latency period in ganglionic neurons, VZV can reactivate and cause herpes zoster (HZ). The latter is favored by advanced age or immunosuppression [1–3] but can also occur in immunocompetent individuals [4–8]. Reactivation of VZV may be accompanied by nervous system complications at both central (meningitis, encephalitis, vasculitis, cerebellitis, myelitis) and/or peripheral levels (cranial nerve palsies, radiculitis) [2, 9]. The clinical symptoms, as well as complementary investigations (blood tests, imaging and electrophysiology), determine the neurological complications. Detecting VZV by real-time polymerase chain reaction (RT-PCR) in skin lesions confirms HZ diagnosis [10], while detection of VZV deoxyribonucleic acid (DNA) in cerebrospinal fluid (CSF) by RT-PCR or identification of intrathecal antiviral antibodies [2], indicates extension of the infection to the nervous system [11, 12]. Antiviral therapy is effective against VZV complications, specifically oral treatment (acyclovir or its pro-drug valacyclovir with better oral absorption) in benign cases and intravenous (IV) acyclovir for severe conditions such as ocular and central nervous system (CNS) complications, especially in immunosuppressed individuals [2, 3].

Here we report the case of an alcohol-dependent older man with zoster paresis in the context of a VZV-associated meningoradiculitis. This clinical entity is not precisely defined in the literature and clear management guidelines about oral versus IV antiviral therapy are scant [13]. Clearly, preventing severe HZ neurological complications should be balanced by careful analysis of the most effective, efficient and least harmful treatment course. We suggest a definition of meningoradiculitis that is usable in clinical practice, including the choice of antiviral therapy. Alcohol- and age-induced cell-mediated immunity (CMI) dysfunction is identified as a plausible precipitating factor of VZV reactivation in our patient.

## Case presentation

A 74-year-old Caucasian man was admitted to the Emergency Room, addressed by his general practitioner for a 3-day history of progressive lower left limb weakness. He complained of a non-traumatic lumbar pain since 10 days. Shortly after, a skin rash appeared on his lower back and extended to the left lower limb. He did not have fever or other new neurological complaints (in particular there was no urine or bowel retention or incontinence). His medical history showed arterial hypertension (treated by an angiotensin converting enzyme and calcic inhibitor); alcohol dependence (with macrocytosis and lower limb polyneuropathy) and benign prostate hypertrophy managed using tamsulosin. Otherwise, he took Aspirin Cardio® and tramadol.

In addition to lower limb peripheral length-dependent abnormalities (ankle jerk areflexia, distal touch and vibration hypoesthesia), the initial neurological assessment revealed significant weakness in hip flexion (M3) and foot dorsiflexion (M2), absence of the left patellar reflex and disturbed position sense on the lower left limb. The Leri sign was positive on the left side. On general examination, clusters of small erythematous vesicular lesions were present on the anterior and internal sides of his left thigh and upper leg. A few similar lesions were also seen on the left part of the lower back. In summary, the clinical picture of the patient showed left L3–L4 sensory-motor deficit associated with radicular zoster. A lumbar computerized tomography (CT)-scan excluded local compression (there was only degenerative lumbar discopathy) and the skin smear was positive for VZV DNA but negative for Herpes simplex virus 1 and 2. The lumbar puncture (see Table 1), despite being traumatic, showed elevated white blood cells, almost exclusively lymphocytes, with high protein levels, blood brain barrier alteration, but no local production of immunoglobulin. Viral activity was detected in the CSF (positive VZV RT-PCR). Electrophysiological studies confirmed left L3–L4 motor and sensory radiculitis with axonotmesis and, as expected, a severe sensori-motor axonal lower limb polyneuropathy. Increased leucocytes and C-reactive protein evidenced mild systemic inflammation; the mean corpuscular volume and IgA levels were slightly increased whereas the thrombocyte rate was low (see Table 1 for more details and reference values). Liver function was normal, as well as thyroid stimulating hormone (TSH) and vitamin B12 and B9 levels. Viral (human immunodeficiency virus, HIV and hepatitis B virus, HBV), Borrelia burgdorferi and Treponema pallidum serology tests were all negative. At this point diagnosis of VZV meningoradiculitis was established.

**Table 1:** Relevant abnormal blood and cerebrospinal fluid results; normal ranges and units (in [])

	Results [normal range and units]
Blood	
Leucocytes	12.3 [4.0–10.0 G/l]
C-reactive protein	18.4 [<5 mg/l]
Thrombocytes	146 [150–350 G/l]
Mean corpuscular volume	101 [80–100 fl]
Immunoglobulin A	3.98 [1.03–3.30 g/l]
Cerebrospinal fluid	
Erythrocytes	139 [0/μl]
White blood cells	139 [0–4/μl] (99.5 % lymphocytes)
Proteins	960 [150–450 mg/l]
Varicella zoster virus polymerase chain reaction	Positive

It should be noted that the patient did not receive zoster vaccine. He was treated within the first 24 h with oral acyclovir followed by IV acyclovir (10 mg/kg every 8 h/ day for 10 days) and showed mainly motor improvement (mild psoas and tibialis anterior paresis finally scored to M4). A few days after admission he developed postherpetic neuralgia that improved with pregabaline and subsequent amitriptyline and a fentanyl patch. Unfortunately analgesic overtreatment led to acute encephalopathy (normal blood tests, brain magnetic resonance imaging and electroencephalogram) that was reversed with dose adjustment. The patient was then transferred to a stationary neurorehabilitation center before returning home.

## Discussion and conclusion

The patient described here had zoster paresis associated with mild inflammation and VZV activity in the CSF; leading to treatment mainly by IV acyclovir. The very few reported cases of VZV meningoradiculitis in the literature can be divided into two groups. The first corresponds to radiculitis without meningitis symptoms (a total of 5 cases of which only 2 were referred to as meningoradiculitis) [14–16]. The second group consists of patients with clinically diagnosed meningitis (or encephalitis) associated with neurological peripheral deficits (only one of the two cases was explicitly mentioned as meningoradiculitis) [4, 7]. Thus, the authors consider meningoradiculitis either to be a purely peripheral (parainfectious?) phenomenon [3, 15] or a CNS complication of VZV infection [2]. Surprisingly, the CSF workup, when available, in these cases similarly displayed mild inflammation [4, 7, 14, 16]. Furthermore, all patients with CSF results were treated by IV antiviral drug regardless of the presence of CNS symptoms or not, and without considering their immune status. In contrast, the 3 cases reported by Chan et al. (no CSF results available) only received supportive treatment (oral acyclovir was not yet available) [15] and yet the outcome did not differ significantly from the other reported cases. Thus, there are obviously contradictions in the definition and management of VZV meningoradiculitis. It appears from the literature that clinicians tend to prescribe IV antiviral treatment to patients with VZV radiculitis after performing CSF investigations, even if this choice does not clearly influence the prognosis.

If meningoradiculitis comprises clinical meningitis with peripheral nerve root involvement, then it should be treated, as with any other VZV-CNS complication, using IV antiviral drugs after spinal tap and other necessary investigations [2, 6, 12]. Alternatively, we suggest considering radiculitis without obvious meningitis symptoms to be a VZV *peripheral* nervous system complication [15]. Such patients should therefore be exempt from CSF workup and, as for most cases in everyday clinical practice (contrary to published cases), orally treated with acyclovir with generally good outcome [2, 3]. The VZV peripheral nervous system complications are most probably underreported if we compare a rate of 3–5 % of herpes zoster [7, 15] to the very low number of reported cases (which are therefore not representative of daily practice as mentioned above). What diagnostic value therefore should inflammatory CSF (with direct or indirect markers of VZV viral activity in the CSF) be given in VZV radiculitis, taking into account that inflammatory markers in the CSF are similar in patients with herpes-related clinical meningitis or encephalitis to those with meningoradiculitis (see above) [12, 14, 16]? Moreover, mild inflammation in the CSF is reported in up to half of all patients with HZ and the presence of viral activity in the CSF does not necessarily establish a causal relationship with neurological symptoms [3]. Along the same lines, in our patient, VZV RT-PCR was positive in the CSF without intrathecal production of immunoglobulin G (IgG) and local contamination from skin lesions in the lumbar region cannot be ruled out. Thus, CSF inflammation should not be used apart from the clinical context as a discriminating criterion between peripheral and central nervous system complications of VZV and should not solely determine the choice of the antiviral treatment route. In the cases published, clinicians perhaps over-treated patients by prescribing IV acyclovir without clinically evident VZV-related CNS symptoms associated with the radiculitis. This was most probably done to prevent severe neurological complications even if, as stated above, this reasoning cannot be fully justified.

None of the frequently reported immunosuppressive states (inflammatory rheumatic diseases, immunosuppressive treatments, HIV infection, etc.) triggering VZV reactivation [3] were found in our patient. However, he was in the most vulnerable age group (advanced age) for VZV reactivation and severity [3], due to age-dependent waning of VZV-related CMI [3, 17, 18]. CMI is important for the control of VZV infection. Similarly, he had signs of alcohol abuse (witnessed by macrocytosis, thrombopenia and increased serum IgA [19–21]), another condition that impairs CMI [21]. So in this case, in the absence of any other evident cause of immunosuppression, age and alcoholism were considered the factors lowering CMI and precipitating HZ. Nonetheless, clear guidelines do not exist for the choice of antiviral treatment in the context of such subtle immunosuppression.

In conclusion, the patient had VZV cutaneous infection with radiculitis coincident with mild CSF inflammation. Reactivation of VZV was most probably precipitated by age- and alcohol-induced cell-mediated immunosuppression. He was diagnosed with meningoradiculitis and treated with IV acyclovir, despite the absence of clinical meningitis. In available published cases, we did not find a consensual definition or management scheme for meningoradiculitis. The presence of CNS symptoms or abnormal CSF findings did not allow discrimination of severe from benign cases and these patients were generally IV treated. However, well-designed clinical trials are needed to review severity criteria for patients with VZV-associated (meningo)-radiculitis, to study the efficiency of oral versus IV antiviral treatment and establish clear guidelines for management. Mild elevation of inflammatory markers in the CSF is not specific or sensitive enough to establish a diagnosis of meningitis without evocative clinical symptoms. Therefore we suggest that until otherwise demonstrated, the term “meningoradiculitis” should be used for and IV antiviral drugs prescribed to patients with clinical meningitis associated with neurological peripheral deficit in the context of VZV infection. Patients with isolated VZV radiculitis should be exempt from CSF investigations and be given oral antiviral treatment, unless they are subject to any condition or pathology decreasing immunity, in particular CMI, in which case CSF workup and IV antiviral therapy may be necessary.

## Abbreviations

CMI: cell-mediated immunity
CNS: central nervous system
CSF: cerebrospinal fluid
CT: computerized tomography
DNA: deoxyribonucleic acid
HBV: hepatitis B virus
HIV: human immunodeficiency virus
HZ: herpes zoster
IgG: immunoglobulin G
IV: intravenous
RT-PCR: real-time polymerase chain reaction
TSH: thyroid-stimulating hormone
VZV: varicella zoster virus
